# Successful Remission of Refractory Oral Ulcers Treated with Low-Dose Thalidomide and Colchicine: A Case Report

**DOI:** 10.3390/reports9010036

**Published:** 2026-01-26

**Authors:** Shun-Yu Kan, Yu-Kai Sung, Chia-Lu Hsu, Kuo-Chou Chiu

**Affiliations:** 1 School of Dentistry, National Defense Medical University, Number 161, Section 6, Minquan East Road, Neihu District, Taipei City 114, Taiwan; michaelkan55555@gmail.com; 2 Department of Pathology, Tri-Service General Hospital, Number 325, Section 2, Chenggong Road, Neihu District, Taipei City 114, Taiwan; adhd057447@gmail.com; 3 School of Pharmacy, National Defense Medical University, Number 161, Section 6, Minquan East Road, Neihu District, Taipei City 114, Taiwan; hsuchiachialu@gmail.com; 4 Department of Dentistry, Taichung Armed Force General Hospital, Number 348, Section 2, Chungshan Road, Taiping District, Taichung City 411, Taiwan

**Keywords:** refractory oral ulcers, aphthous stomatitis, thalidomide, colchicine, combination therapy

## Abstract

**Background and Clinical Significance**: Oral ulcers are a common disease for dental practitioners. The policy of treating oral ulcers includes removing etiology and medication. Standard management of oral ulcers includes elimination of etiologic factors and pharmacologic therapy. Topical corticosteroids are the most commonly used medicine for oral ulcers. Exclude possible etiologies related to ulcers; refractory ulcers need systemic evaluation and precise medication use to improve patients’ quality and satisfaction. **Case Presentation**: We present a case of refractory oral ulcers resistant to multiple conventional treatments, which were found to be ineffective. These ulcers significantly impact patient quality of life. We prescribed a series of oral ulcer treatments following the removal of cause factors, such as rounding the teeth and making a soft occlusal bite plate to reduce traumatic sources from the patient’s Parkinson’s disease. A biopsy of the ulcer lesions was also done. All the treatments involving corticosteroids and removing the ulcer-associated etiology were ineffective. **Conclusions**: Finally, combined therapy using low-dose thalidomide (50 mg/day) and colchicine (1.5 mg/day) resulted in substantial clinical improvement, and complete remission was sustained for over six months without recurrence. A narrative discussion of relevant literature is provided to contextualize therapeutic considerations in refractory oral ulceration. Conclusion: This case suggests that low-dose thalidomide and colchicine combination therapy may be a therapeutic consideration for refractory oral ulcers when conventional management fails; however, the observation is hypothesis-generating and further studies are required to evaluate efficacy and safety.

## 1. Introduction and Clinical Significance

Oral ulcers, a common condition in oral mucosal pathology, include recurrent aphthous stomatitis (RAS) and traumatic ulcers as the primary types. While most ulcers heal naturally, refractory cases significantly impact patient comfort and life quality. Standard management involves removing related cause factors and prescribing topical or systemic corticosteroids. In challenging cases, subcutaneous corticosteroid injections have been recommended [1,2]. However, anecdotal studies have suggested that dapsone, pentoxifylline, colchicine, and thalidomide may have a role in refractory cases [3,4,5]. These studies often involved single-dose medication for refractory ulcers. Accurate diagnosis and eliminating potential cause factors are crucial initial steps in managing oral ulcers. Biopsy of prolonged unhealed oral ulcers is advised to rule out possible malignancies. Here, we report a case of refractory oral ulcers managed with combination therapy using low-dose thalidomide and colchicine, aiming to provide clinical insight in a refractory setting.

## 2. Case Presentation

**Figure 1:**
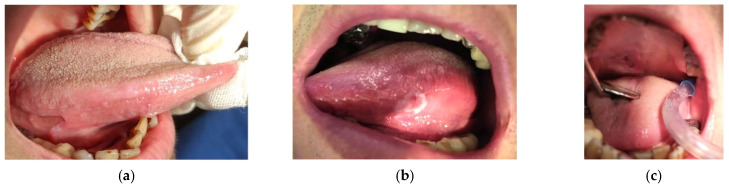
Initial intraoral examination revealed multiple ulcerative lesions and marked dryness of oral mucosa. (**a**) Ulceration on the right lateral border of the tongue (2.5 × 1 cm). (**b**) Ulceration observed on the left lateral border of the tongue (1.5 × 1 cm). (**c**) Ulcerative lesion located on the soft palate (1 × 1 cm).

**Figure 2:**
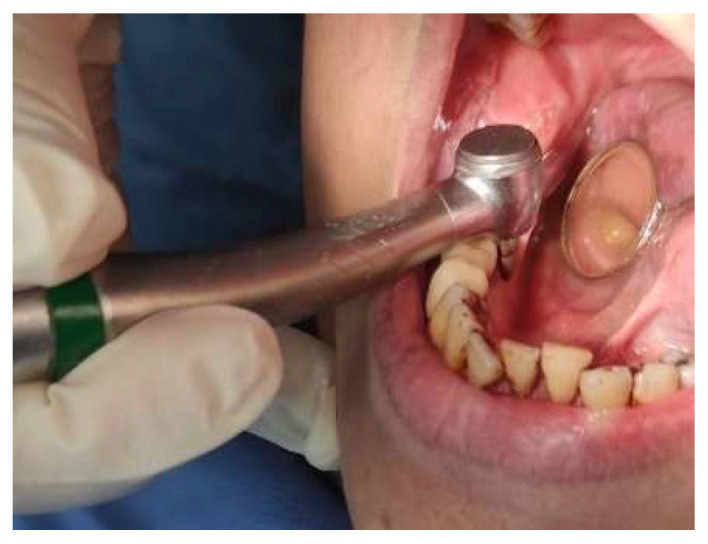
Sharp mandibular posterior teeth edges were smoothed and reshaped into rounded contours to minimize mechanical trauma.

**Figure 3:**
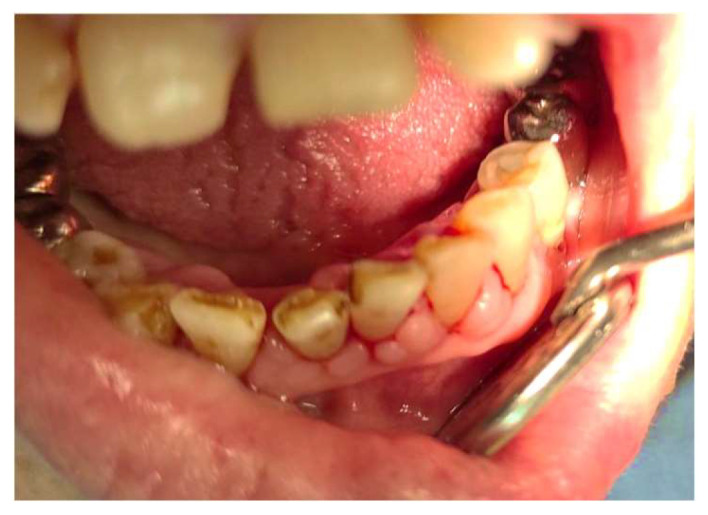
Generalized gingival redness and swelling noted throughout the dentition, associated with plaque deposition.

A 66-year-old female presented to our clinic with persistent painful oral ulcerations lasting more than two weeks. Her medical history included Sjögren’s syndrome, systemic lupus erythematosus (SLE), rheumatoid arthritis, Parkinson’s disease, and impaired liver function. The patient denied smoking, alcohol consumption, or betel nut chewing. Given the presence of multiple systemic autoimmune diseases, these conditions were considered potential confounding factors in both disease severity and treatment response. Clinical examination revealed three distinct ulcerations located on the right lateral tongue (2.5 × 1 cm, Figure 1a), left lateral tongue (1.5 × 1 cm, Figure 1b), and soft palate (1 × 1 cm, Figure 1c). Sharp tooth structures over lower bilateral molar areas with Xerostomia were also noted (Figure 2). Generalized gingival swelling with plaque accumulation was also observed (Figure 3). Baseline pain intensity was retrospectively assessed using a visual analog scale (VAS, 0–10) and was estimated to be 8, reflecting severe pain interfering with oral intake and speech.

### 2.1. Initial Management and Etiologic Control

**Figure 4:**
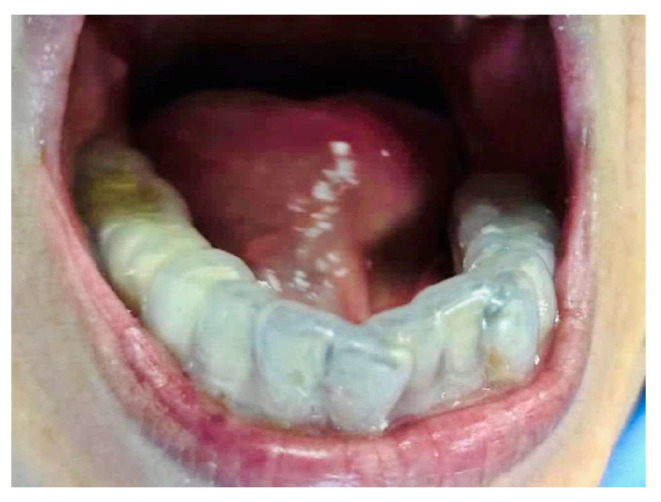
A soft occlusal splint was fabricated and placed to minimize frictional trauma due to involuntary tongue movements exacerbated by xerostomia associated with Parkinson’s disease.

Initially, bilateral tongue ulcers were attributed to mechanical trauma from sharp teeth combined with Xerostomia. In contrast, the palatal ulcer was considered to be associated with her underlying autoimmune condition. Our treatment plan was comprehensive, beginning with the meticulous task of smoothing sharp tooth edges and the precise application of dexamethasone (1 mg/g) oral base topically. However, after two weeks, the ulcers demonstrated minimal improvement. Considering Parkinson’s disease-related tongue movements and persistent Xerostomia, we made a soft occlusal bite splint and prescribed oral balance gel for mucosal lubrication (Figure 4). Concurrently, we prescribed topical dexamethasone (1 mg/g) oral base, benzydamine oral spray (3 mg/mL), and tramadol (375 mg twice daily) for symptomatic relief. Routine scaling was performed regularly, yet the ulcers persisted with limited improvement after four weeks. VAS scores during this period remained high (VAS 7–8), with no meaningful reduction in ulcer size.

### 2.2. Histopathological Evaluation and Systemic Therapy

**Figure 5:**
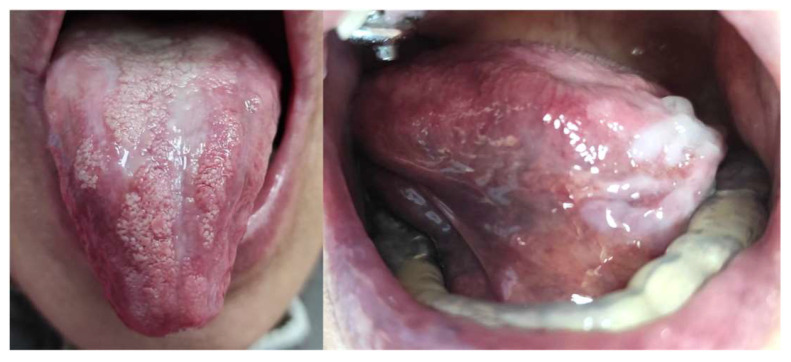
Presence of pseudomembranous candidiasis presenting as white plaques on oral mucosa, indicating secondary fungal infection due to prolonged use of corticosteroids.

**Figure 6:**
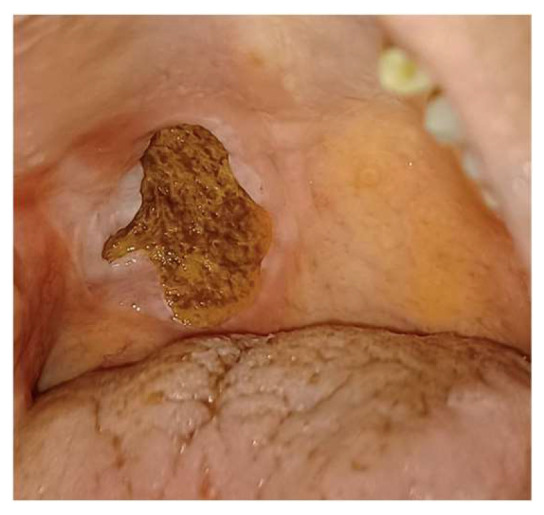
Persistent ulceration on the right side of the soft palate measuring approximately 2.3 × 2.3 cm, with yellow-brown mucosal pigmentation secondary to the therapeutic use of nystatin mouthwash.

An excisional biopsy from the left lateral tongue was conducted at the six-week follow-up to rule out malignancy. The pathological report shows epithelial hyperplasia with no malignant transformation. Subsequent management included systemic prednisolone (initially 20 mg/day for 1 week, then reduced to 10 mg/day) with nystatin mouth rinse (100,000 I.U./c.c, thrice daily) to manage pseudomembranous candidiasis (Figure 5). Steroid resistance was clinically defined as the persistence of ulcer size and pain despite adequate courses of topical and systemic corticosteroids. The patient had a history of intermittent systemic corticosteroid exposure related to her autoimmune diseases, although she was not receiving continuous long-term steroid therapy at presentation. Despite the initial treatment not yielding the expected results, the patient’s resilience was evident as the ulcer on the right soft palate showed minimal improvement, measuring 2.3 × 2.3 cm (Figure 6). Even after the addition of thalidomide (50 mg/day) combined with prednisolone (10 mg/day), the ulcer on the right soft palate showed minimal improvement, measuring 2.3 × 2.3 cm after one-week follow-up (Figure 6). With VAS remaining approximately 7. The short duration of monotherapy was not intended to establish definitive drug inefficacy but rather served as an exploratory assessment of early clinical responsiveness and tolerability in the setting of severe symptoms and multiple systemic comorbidities.

### 2.3. Escalation and Combination Therapy

**Figure 7:**
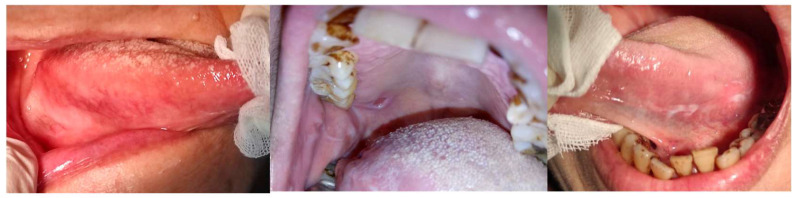
Partial improvement observed in oral ulcers following combined treatment with thalidomide, colchicine, and prednisolone, though complete resolution was not initially achieved.

**Figure 8:**
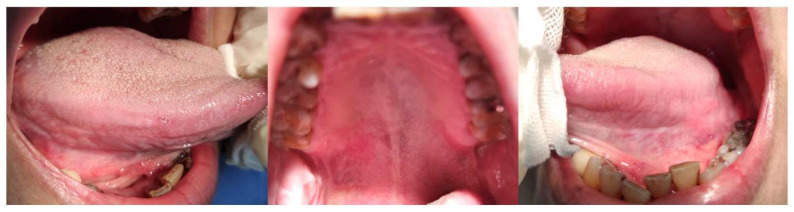
Significant clinical improvement of oral ulcers following optimized combined therapy with thalidomide (50 mg/day), colchicine (0.5 mg three times daily), and low-dose prednisolone (2.5 mg/day). Ulcers substantially decreased in size, number, pain intensity, and frequency of episodes.

Colchicine (0.5 mg twice daily) was introduced to replace thalidomide temporarily, and the prednisolone dose was raised to 15 mg the following week; however, ulcer progression was limited, with persistent pain and stable lesion size (VAS ~6–7). A combination treatment regimen involving thalidomide (50 mg/day), colchicine (1.5 mg/day), and low-dose prednisolone (2.5 mg/day) was subsequently initiated. Within two weeks, notable pain reduction was observed (VAS decreased from 7 to approximately 3), accompanied by progressive reduction in ulcer size (Figure 7 and Figure 8). During brief discontinuation of either thalidomide or colchicine due to clinical considerations, ulcer pain and mucosal breakdown recurred within several days, with VAS increasing to approximately 6, and improvement was again observed after resumption of combination therapy. Optimizing the regimen with thalidomide (50 mg/day), colchicine (1.5 mg/day), and prednisolone (2.5 mg/day) achieved sustained ulcer control for the following two weeks. Prednisolone was subsequently discontinued, while thalidomide and colchicine were continued. No ulcer recurrence or pain exacerbation (VAS ≤1) was observed during the subsequent six weeks. A final maintenance regimen combining thalidomide (50 mg/day) with colchicine (1.5 mg/day) resulted in complete remission, which was maintained throughout a six-month follow-up period without recurrence or discomfort.

### 2.4. Histopathological Follow-Up

Biopsies were performed at four follow-up periods to exclude malignant transformation during the disease course.

These repeated biopsies were performed for surveillance rather than to guide therapeutic escalation.

**Figure 9:**
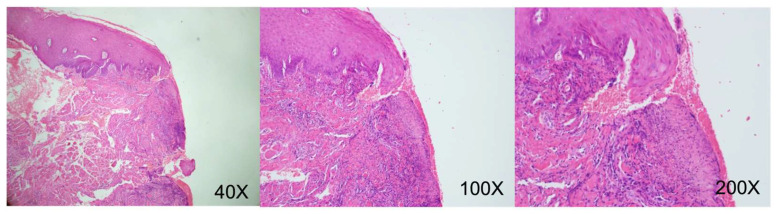
Histopathological findings demonstrate focal full-thickness epithelial loss accompanied by necrotic debris and hemorrhagic exudate, consistent with chronic ulcerative inflammation. (Original uncropped and unadjusted microscopy images for figures are provided as Supplementary Material).

**Figure 10:**
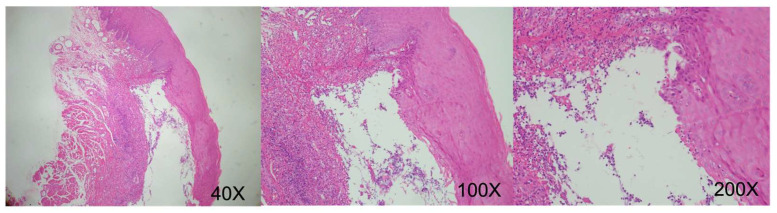
Histological analysis revealing extensive destruction involving mucosal and submucosal layers, characterized by necrotic tissue fragments and infiltration by mixed inflammatory cells extending deeply into the submucosa. (Original uncropped and unadjusted microscopy images for figures are provided as Supplementary Material).

**Figure 11:**
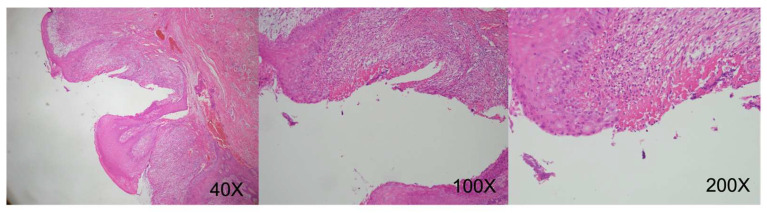
Microscopic examination indicating focal ulceration with architectural distortion of the epithelium, dense chronic inflammatory infiltrates, and prominent submucosal vascular congestion. (Original uncropped and unadjusted microscopy images for figures are provided as Supplementary Material).

**Figure 12:**
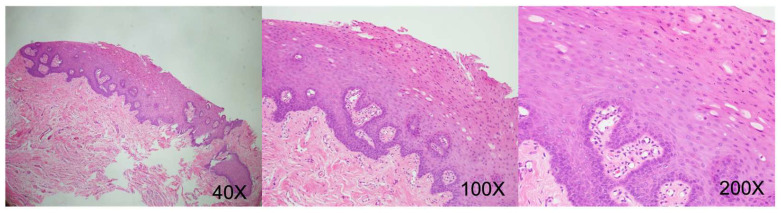
Histopathological examination showing mild epithelial hyperplasia and parakeratosis, mild chronic inflammatory cell infiltration, and granulation tissue formation within the submucosal tissue.

1. August 2019 (right lateral tongue): Squamous epithelial hyperplasia with low-grade dysplasia, focal ulceration. (CK-negative, increased Ki-67 proliferative activity) (Figure 9).
2. December 2019 (left lateral tongue): Ulceration with granulation tissue formation. Deep submucosal inflammatory cell infiltration. (CK-negative) (Figure 10).
3. October 2020 (oral mucosa): Squamous epithelial hyperplasia, focal low-grade dysplasia, chronic inflammation, submucosal congestion. (CK-negative) (Figure 11).
4. April 2024 (right tongue): Mild squamous epithelial hyperplasia, parakeratosis, mild chronic inflammation. (CK-negative and basal Ki-67 proliferative activity) (Figure 12).

## 3. Discussion

Recurrent oral ulcers are classified into minor, major, and herpetiform categories based on size, number, shape, location, healing duration, and the presence or absence of scarring [1,2]. Depending on severity and recurrence frequency, they may also be differentiated into simple or complex aphthosis. Simple aphthosis typically involves smaller lesions associated with mild pain and infrequent recurrences. In contrast, complex aphthosis presents with larger, multiple, and overlapping lesions accompanied by severe pain, prolonged healing periods, and lesions often extending beyond the oral cavity [2]. The extensive and persistent ulcers observed in our patient met the severe and complex aphthosis criteria. The pathogenesis of recurrent oral ulcers remains incompletely understood but likely involves T-cell-mediated mucosal immune dysregulation, exacerbated by cytokine and neuropeptide activity. Although potential etiologies include infections, nutritional deficiencies, and systemic diseases such as Crohn’s disease or celiac disease, these are rarely the primary contributors to most cases [1]. Diagnosis, a process that relies on clinical evaluation and patient history, provides a reassuring framework, supplemented by biopsies when necessary to exclude malignancies and systemic conditions such as Crohn’s disease, systemic lupus erythematosus, Behçet’s disease, pemphigus, pemphigoid, cyclic neutropenia, or squamous cell carcinoma [1,2,6,7,8,9,10,11,12,13,14,15,16,17,18,19,20,21,22,23,24,25].

Corticosteroids, notably prednisolone, are frequently prescribed for severe presentations, are initiated at 20–40 mg/day for approximately one week, and are gradually tapered down as the symptoms improve [1,7]. Although corticosteroids effectively promote acute healing, they do not reliably prevent recurrence and pose risks of systemic side effects, limiting their long-term usage [7]. For refractory cases, alternative pharmacological treatments, including thalidomide, colchicine, dapsone, pentoxifylline, and biological agents such as adalimumab, are recommended. However, most of the reports were single-dose use with no combination therapy [1,2,4,5,26,27,28]. Existing reports primarily describe monotherapy approaches, and evidence regarding combination strategies remains limited.

Thalidomide is considered an anti-angiogenic agent in tumor therapy and an immunomodulatory drug by its pharmacological properties of reducing tumor necrosis factor-alpha (TNF-α) levels to eliminate host inflammatory reactions and tissue damage [7]. Due to the anti-angiogenic properties in the fetuses, the teratogenicity side effects should be considered and avoided in pregnant women or women at puberty periods. Colchicine is an anti-inflammatory drug that disrupts microtubule assembly and reduces neutrophil activity to reduce host inflammatory reactions. Dapsone modulates neutrophil chemotaxis and lysosomal function and should not be prescribed in patients with glucose-6-phosphate dehydrogenase (G6PD) deficiencies or sulfa drug allergies. Pentoxifylline, a drug with the potential to enhance microcirculation and suppress TNF-α production, is an area of ongoing research due to its unclear mechanism [4]. Biological agents, such as adalimumab, have shown efficacy but with higher costs, and there is no oral prescription but only IV injections [29].

Clinical studies indicate that thalidomide at 100 mg/day substantially decreases ulcer frequency and severity; however, recurrence commonly occurs after discontinuation. Low maintenance doses (approximately 50 mg/day) result in promising remission rates but necessitate ongoing safety evaluations. Published clinical trials indicated that thalidomide is more effective in treating oral ulcers than dapsone, colchicine, and pentoxifylline [30,31,32]. Most clinical trials use a single dose of medicine to treat refractory ulcers. Reports evaluating combination immunomodulatory therapy remain scarce, and comparative evidence is limited. The present case provides an additional clinical observation of combination therapy using thalidomide and colchicine in a patient with refractory disease and limited response to corticosteroids. Initiation at lower doses with close monitoring may be considered to mitigate potential adverse effects; however, such strategies require further evaluation.

Clinically validated alternative treatments with broader applications include thalidomide, colchicine, dapsone, and pentoxifylline [4,5]. Revuz et al. demonstrated that nearly half of patients treated with thalidomide (100 mg/day) achieved complete remission within two months; however, ulcer recurrence averaged 19 days post discontinuation [31]. Hello et al. reported that maintenance therapy with low-dose thalidomide (median dose 50 mg/day) effectively maintains remission, yet the risk of recurrence after cessation remains notable, highlighting the need for continuous monitoring [32]. An open clinical trial by Mimura et al. further corroborated the superior effectiveness and tolerability of thalidomide compared with other treatments [5].

This report has several limitations. First, it describes a single patient without a controlled comparison, limiting causal inference. Second, outcome measures such as pain intensity and ulcer size were retrospectively estimated rather than prospectively standardized. Third, the presence of multiple systemic autoimmune diseases and prior corticosteroid exposure represents potential confounding factors. Fourth, although remission was maintained for six months, long-term safety and generalizability of combination therapy remain uncertain. Therefore, the findings should be interpreted cautiously and viewed as hypothesis-generating rather than practice-changing.

## 4. Conclusions

Although combination therapy with low-dose thalidomide and colchicine is not routine therapy with oral ulcers, the cost of combination therapy is ten times that compared with traditional corticosteroid therapy in Taiwan. The teratogenic side effects of using thalidomide should be considered. When considering combination therapy, individual patient factors—including systemic comorbidities, financial burden, and tolerance of potential adverse effects—should be carefully weighed. Overall, this case should be interpreted as hypothesis-generating and does not establish clinical recommendations or generalizable treatment efficacy.

## Data Availability

The datasets generated and/or analysed during the current study are not publicly available due to privacy and ethical considerations but are available from the corresponding author on reasonable request.

## Supplementary Materials

The following are available online at https://www.mdpi.com/article/10.3390/reports9010036/s1, Figures: Histopathological findings.

## Abbreviations

The following abbreviations are used in this manuscript:

RAS	Recurrent aphthous stomatitis
SLE	Systemic lupus erythematosus
