# Supplementary figures and images for: Successful Remission of Refractory Oral Ulcers Treated with Low-Dose Thalidomide and Colchicine: A Case Report
Source: Reports (MDPI). 2026 Jan 26;9(1):36. doi: 10.3390/reports9010036 (PMC12922018; doi:10.3390/reports9010036)

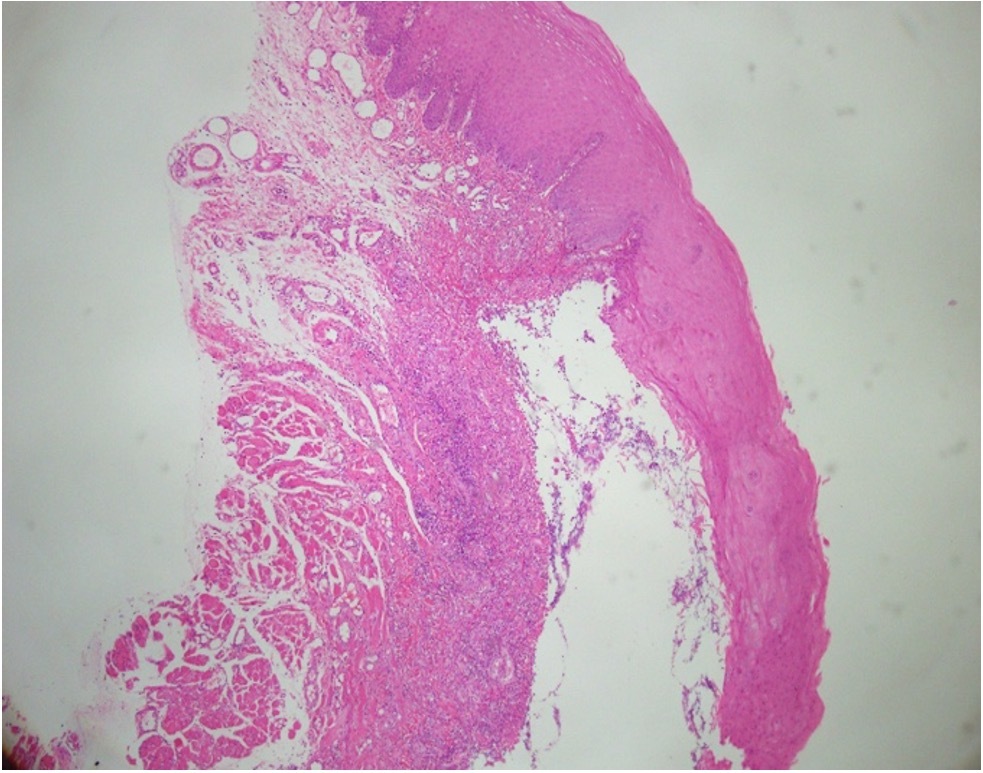

Supplement: Supplementary file 1 [file reports-09-00036-s001.zip › Supplementary Material_/Figure 10-1.jpg]

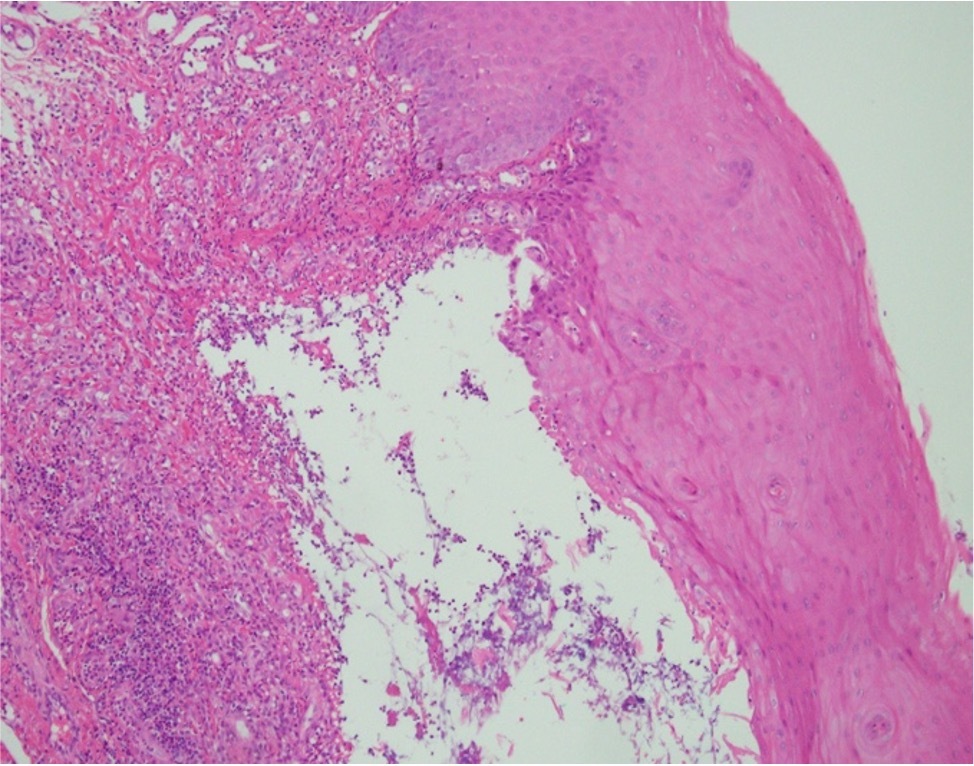

Supplement: Supplementary file 1 [file reports-09-00036-s001.zip › Supplementary Material_/Figure 10-2.jpg]

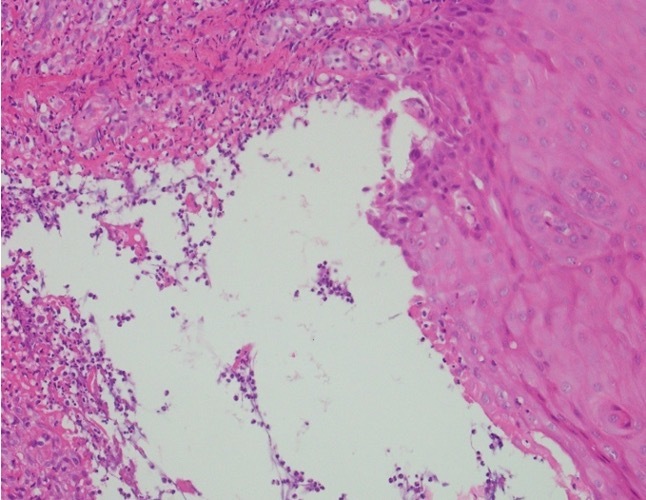

Supplement: Supplementary file 1 [file reports-09-00036-s001.zip › Supplementary Material_/Figure 10-3.jpg]

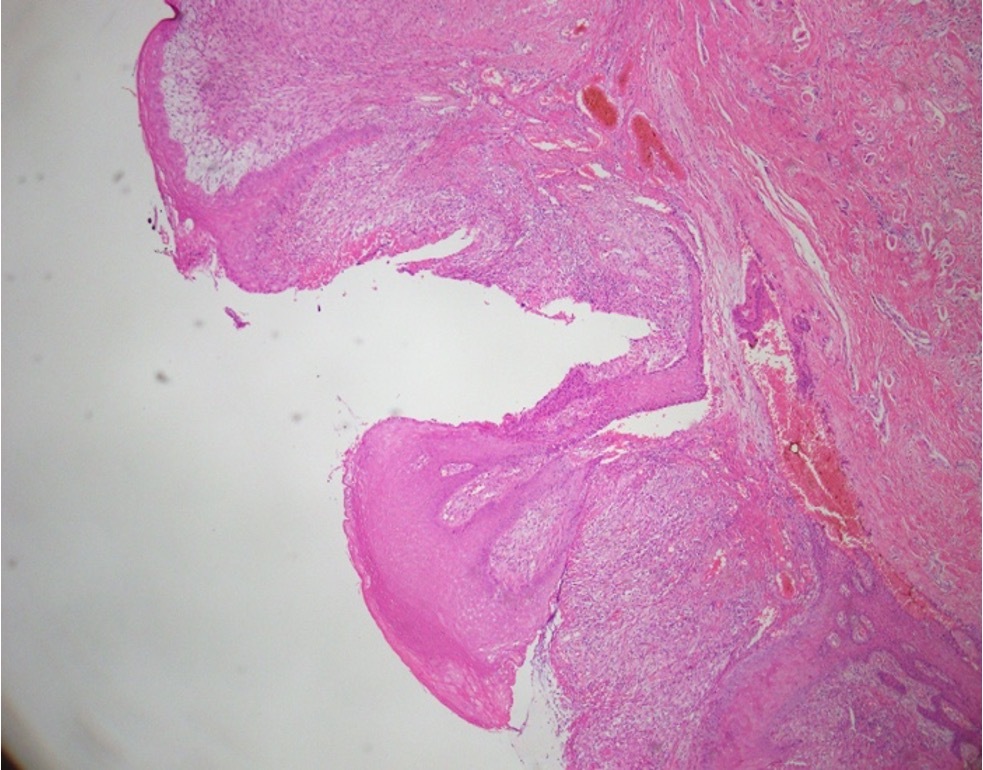

Supplement: Supplementary file 1 [file reports-09-00036-s001.zip › Supplementary Material_/Figure 11-1.jpg]

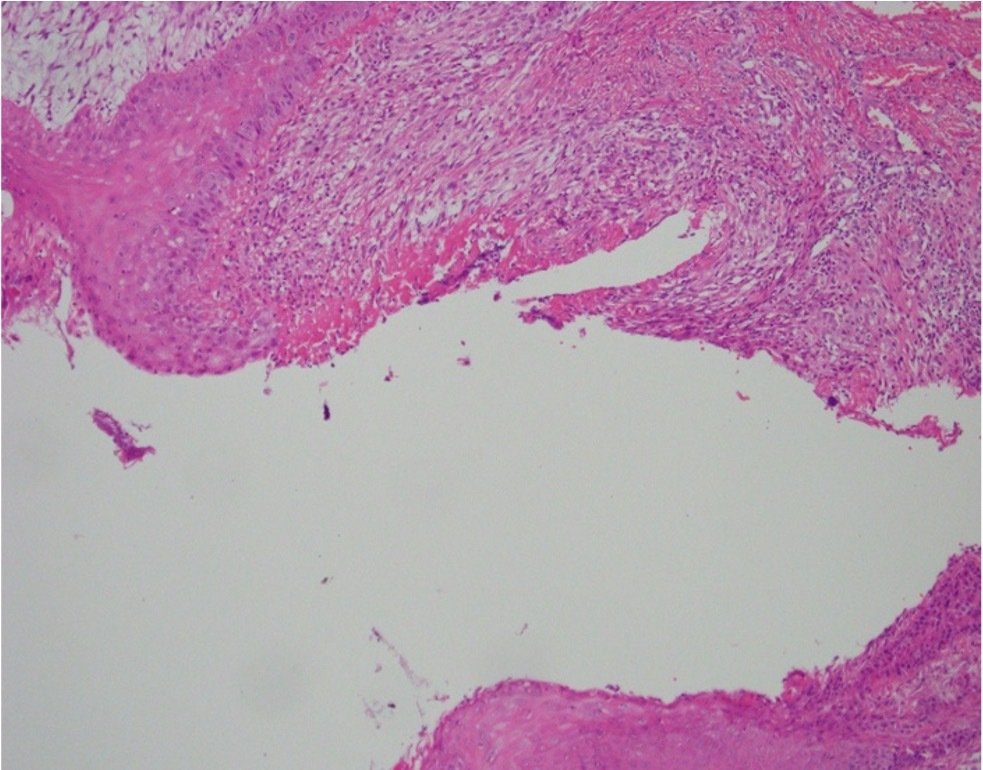

Supplement: Supplementary file 1 [file reports-09-00036-s001.zip › Supplementary Material_/Figure 11-2.jpg]

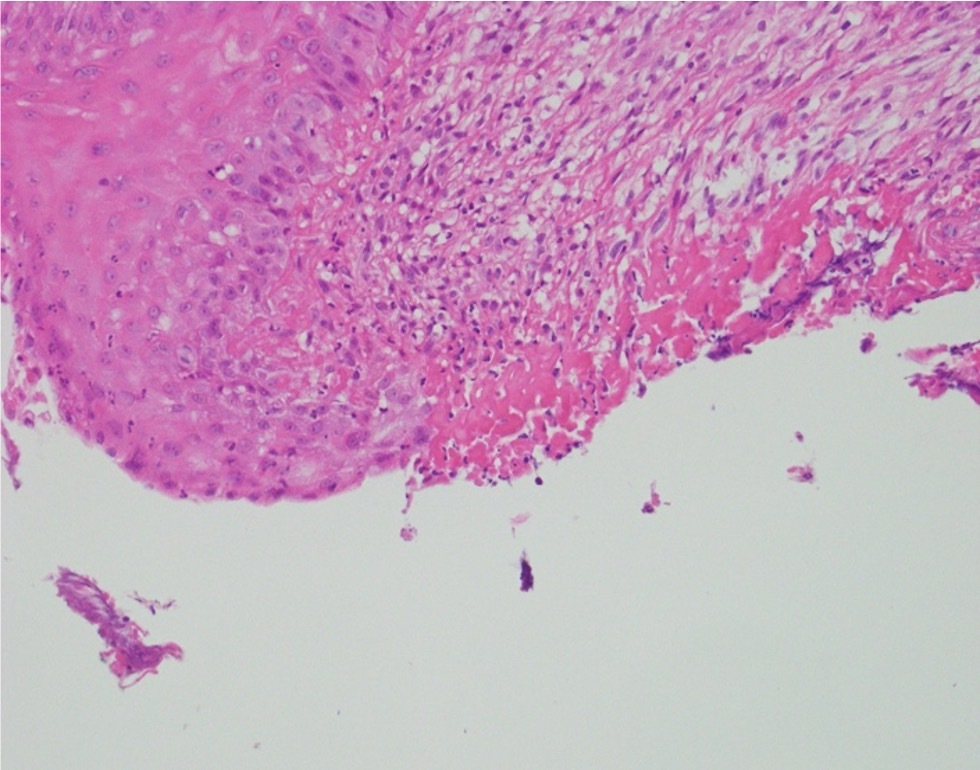

Supplement: Supplementary file 1 [file reports-09-00036-s001.zip › Supplementary Material_/Figure 11-3.jpg]

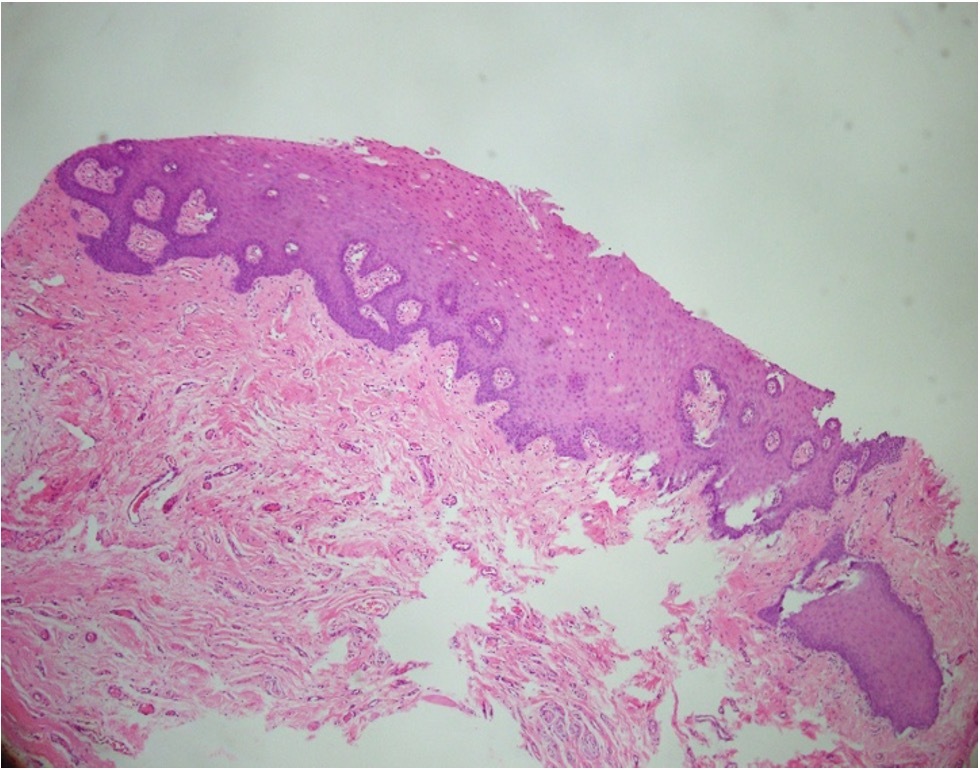

Supplement: Supplementary file 1 [file reports-09-00036-s001.zip › Supplementary Material_/Figure 12-1.jpg]

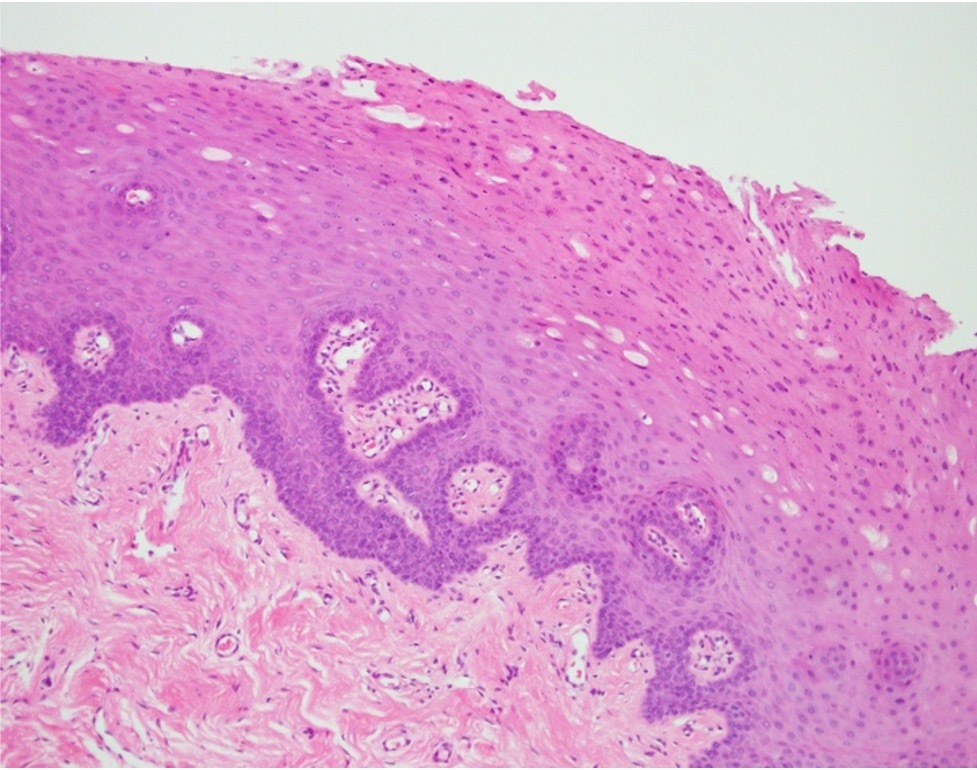

Supplement: Supplementary file 1 [file reports-09-00036-s001.zip › Supplementary Material_/Figure 12-2.jpg]

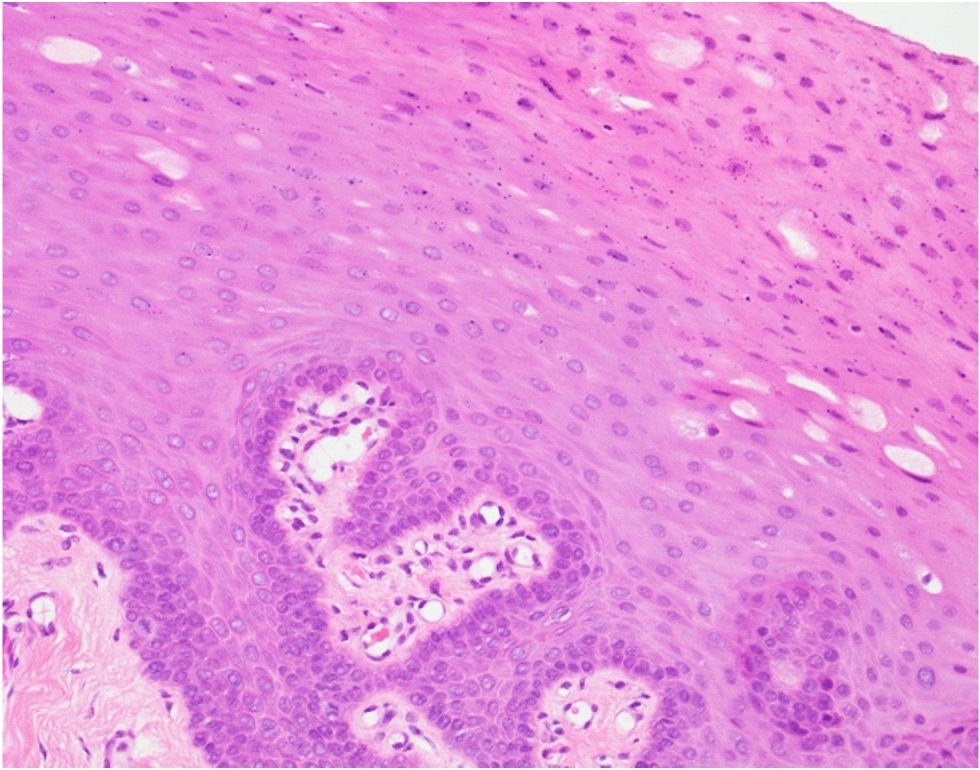

Supplement: Supplementary file 1 [file reports-09-00036-s001.zip › Supplementary Material_/Figure 12-3.jpg]

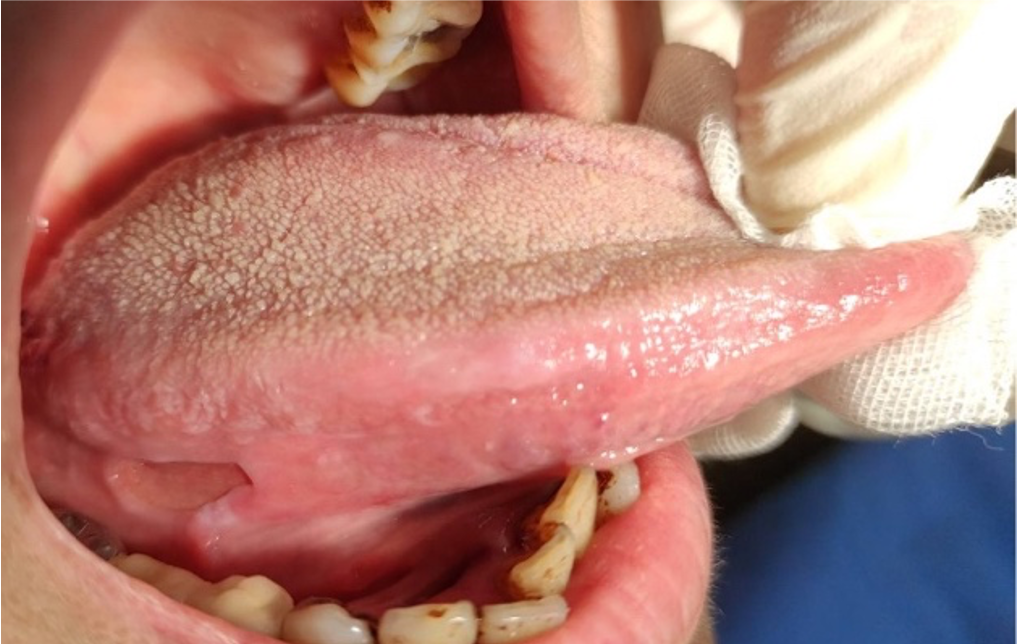

Supplement: Supplementary file 1 [file reports-09-00036-s001.zip › Supplementary Material_/Figure 1a.png]

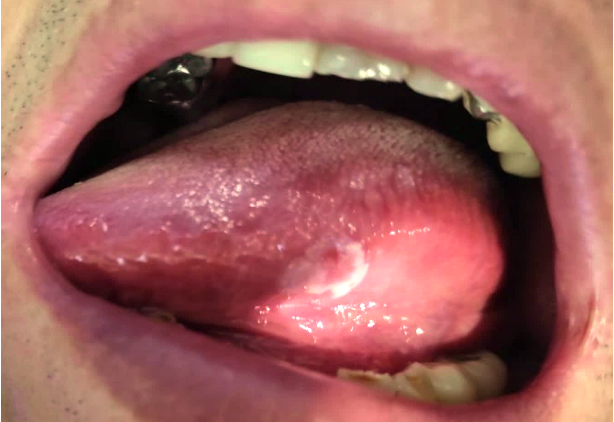

Supplement: Supplementary file 1 [file reports-09-00036-s001.zip › Supplementary Material_/Figure 1b.png]

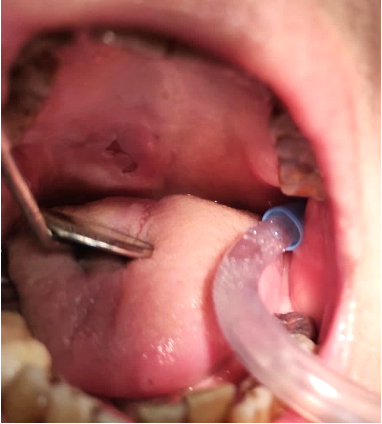

Supplement: Supplementary file 1 [file reports-09-00036-s001.zip › Supplementary Material_/Figure 1c.png]

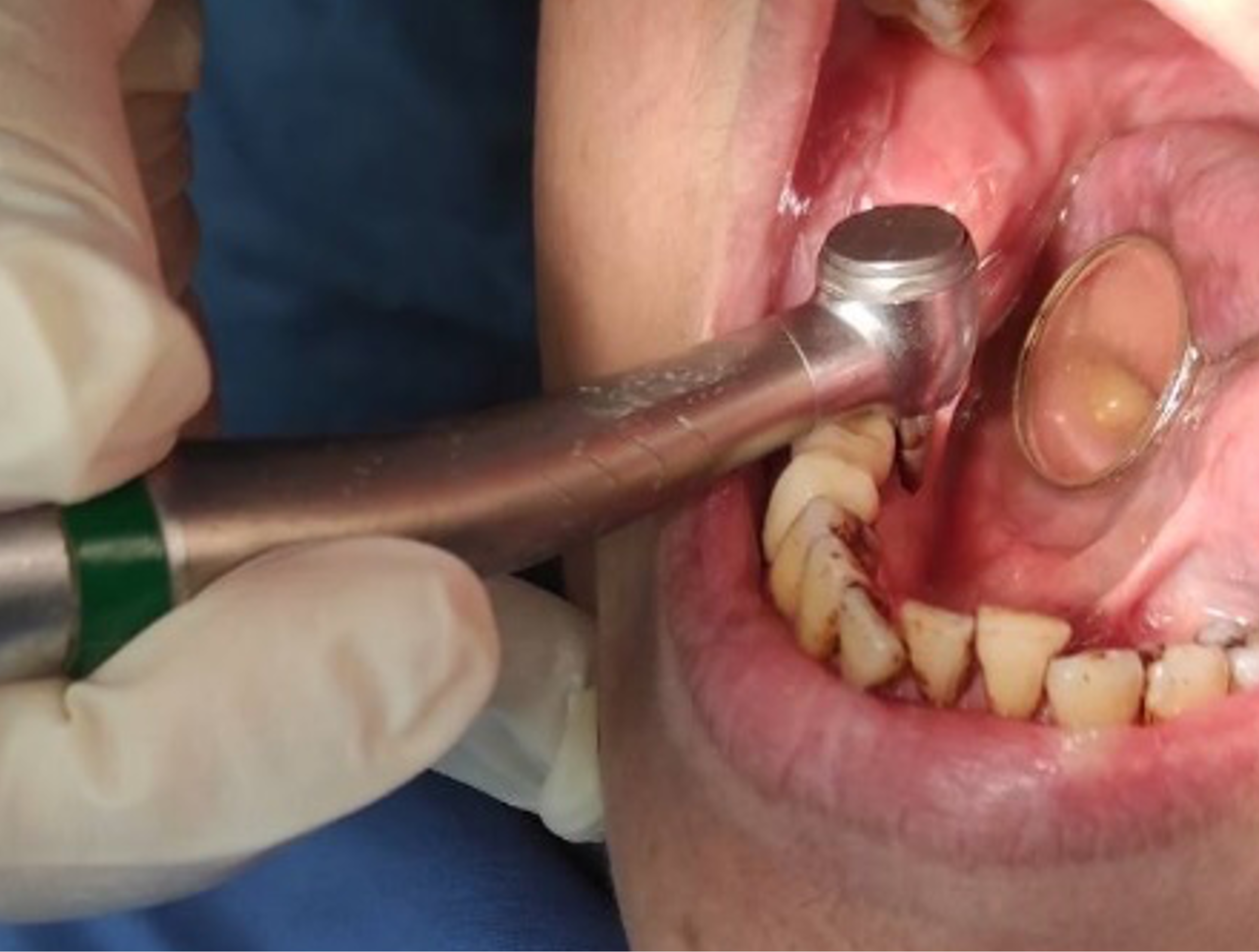

Supplement: Supplementary file 1 [file reports-09-00036-s001.zip › Supplementary Material_/Figure 2.png]

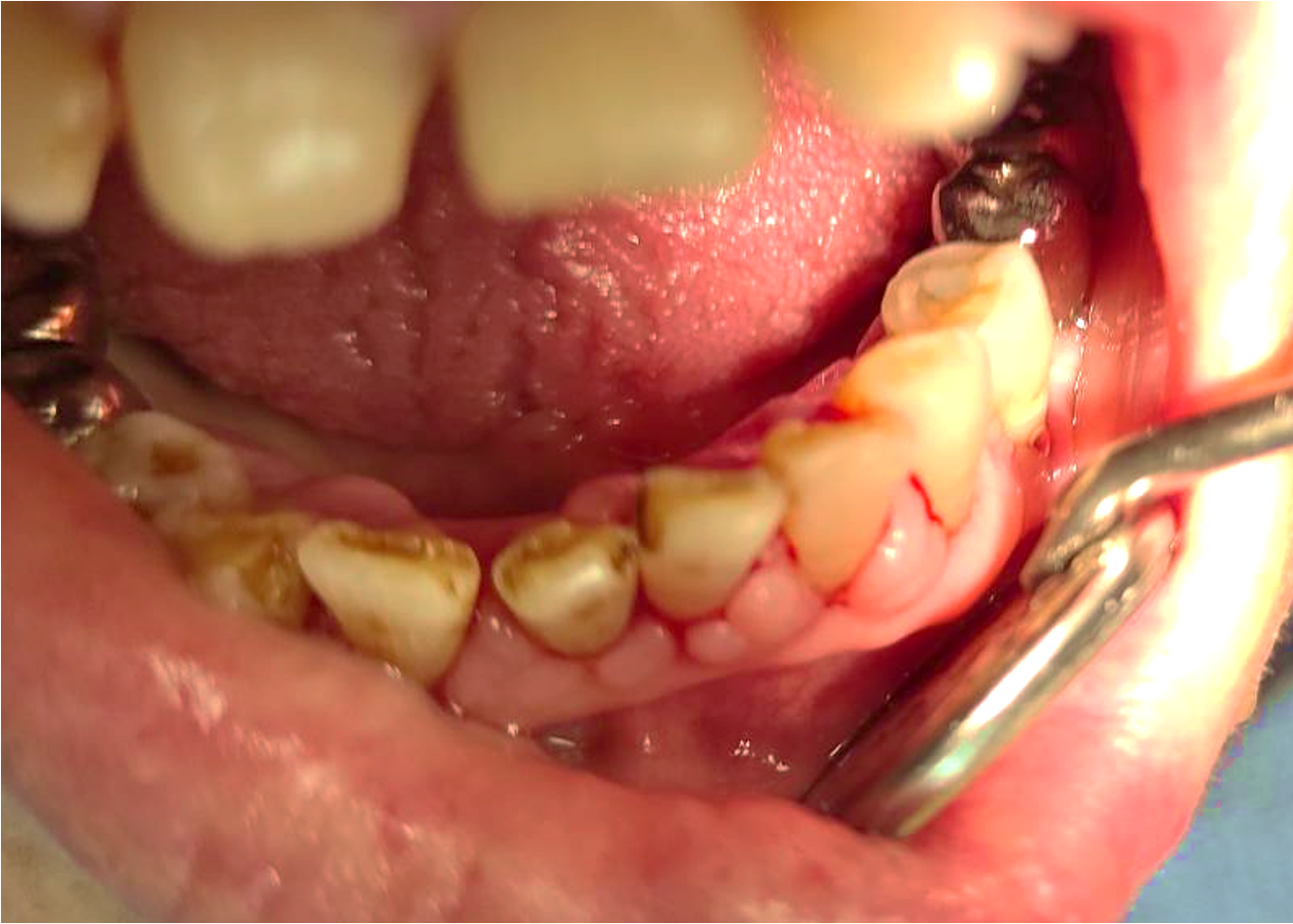

Supplement: Supplementary file 1 [file reports-09-00036-s001.zip › Supplementary Material_/Figure 3.png]

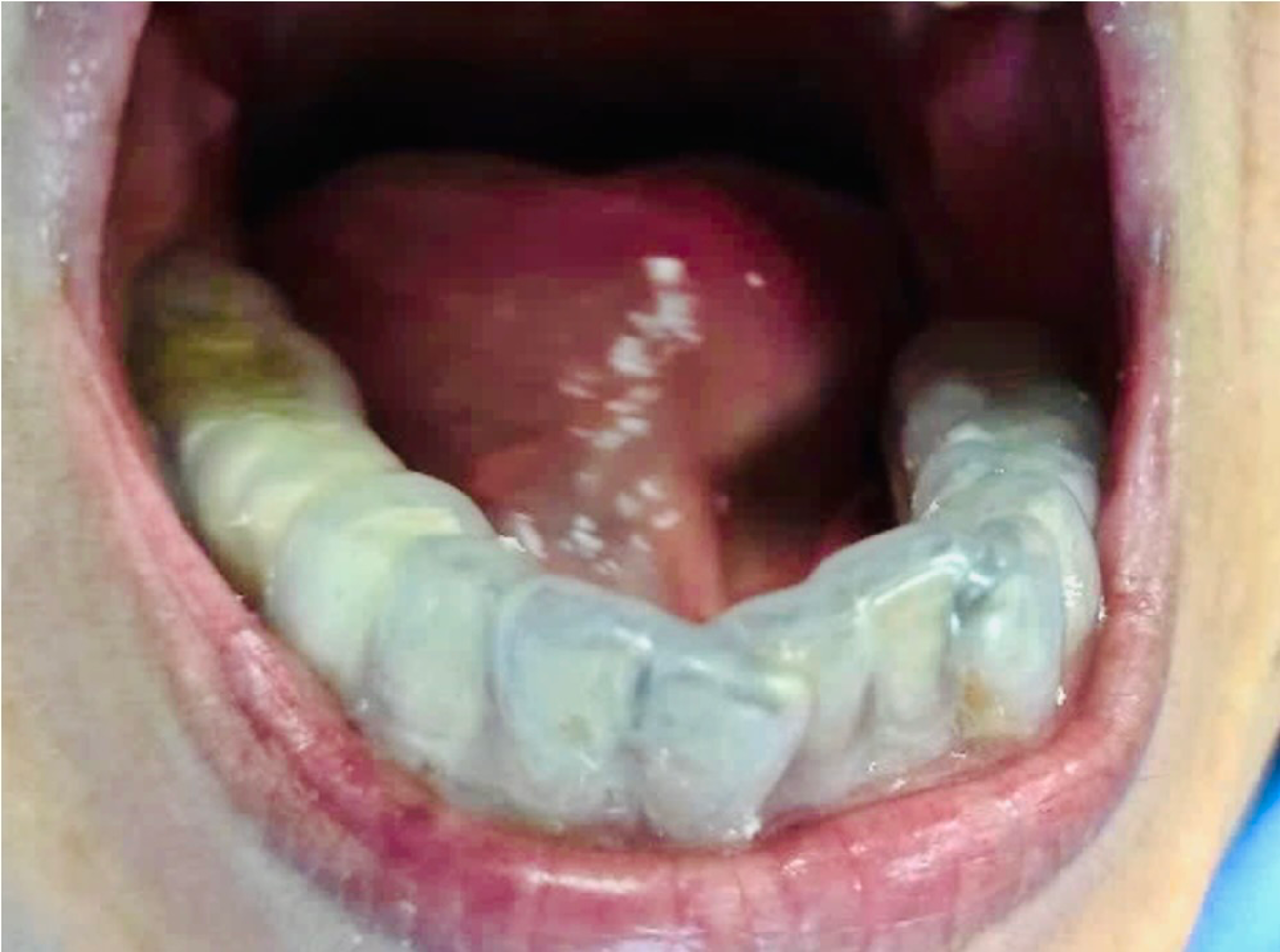

Supplement: Supplementary file 1 [file reports-09-00036-s001.zip › Supplementary Material_/Figure 4.png]

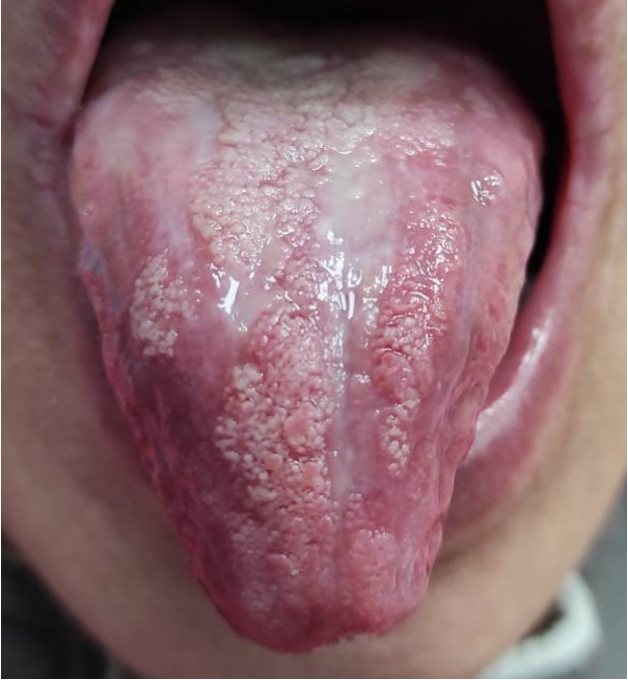

Supplement: Supplementary file 1 [file reports-09-00036-s001.zip › Supplementary Material_/Figure 5-1.png]

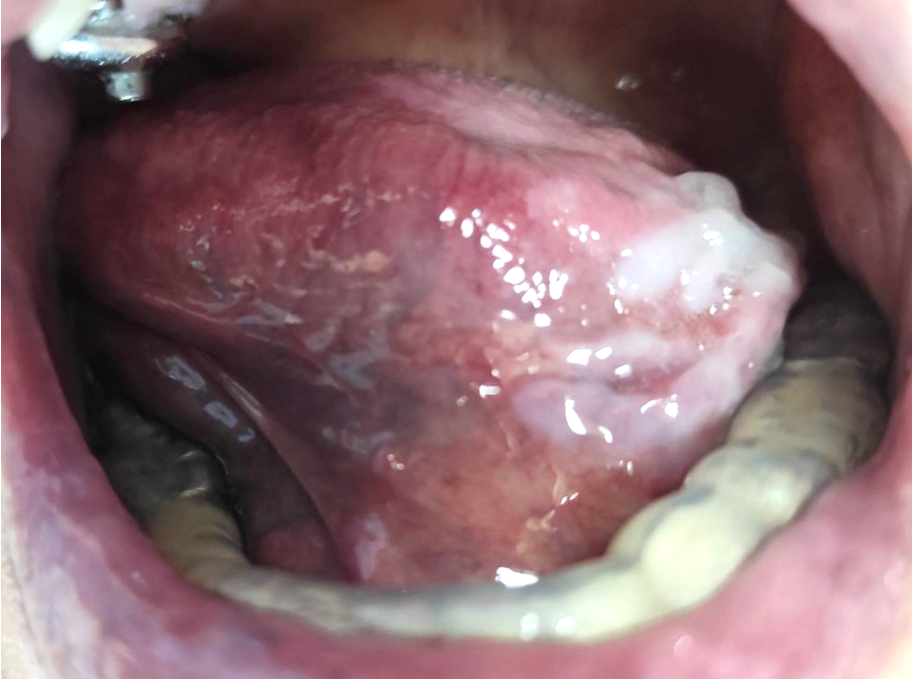

Supplement: Supplementary file 1 [file reports-09-00036-s001.zip › Supplementary Material_/Figure 5-2.png]

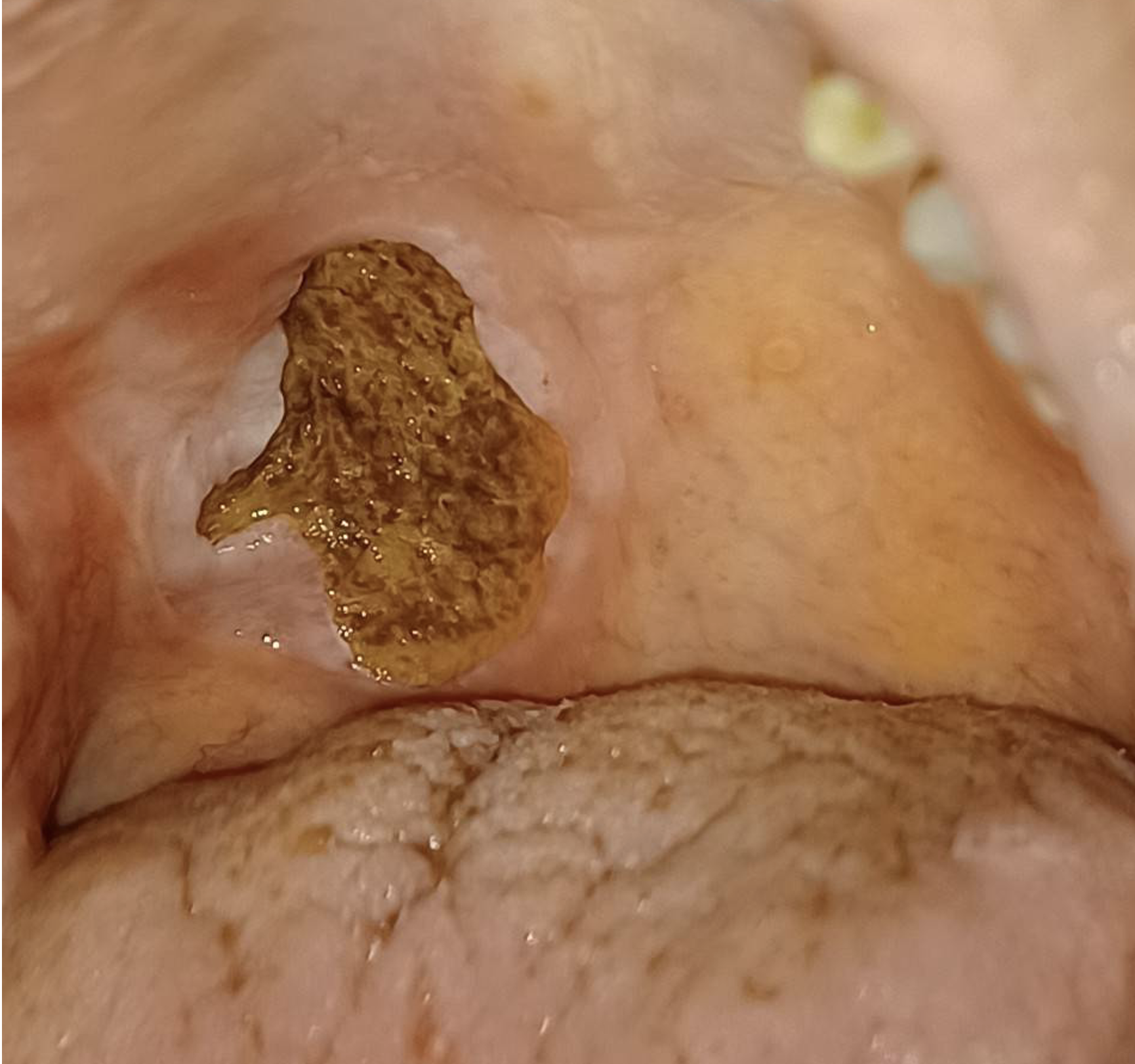

Supplement: Supplementary file 1 [file reports-09-00036-s001.zip › Supplementary Material_/Figure 6.png]

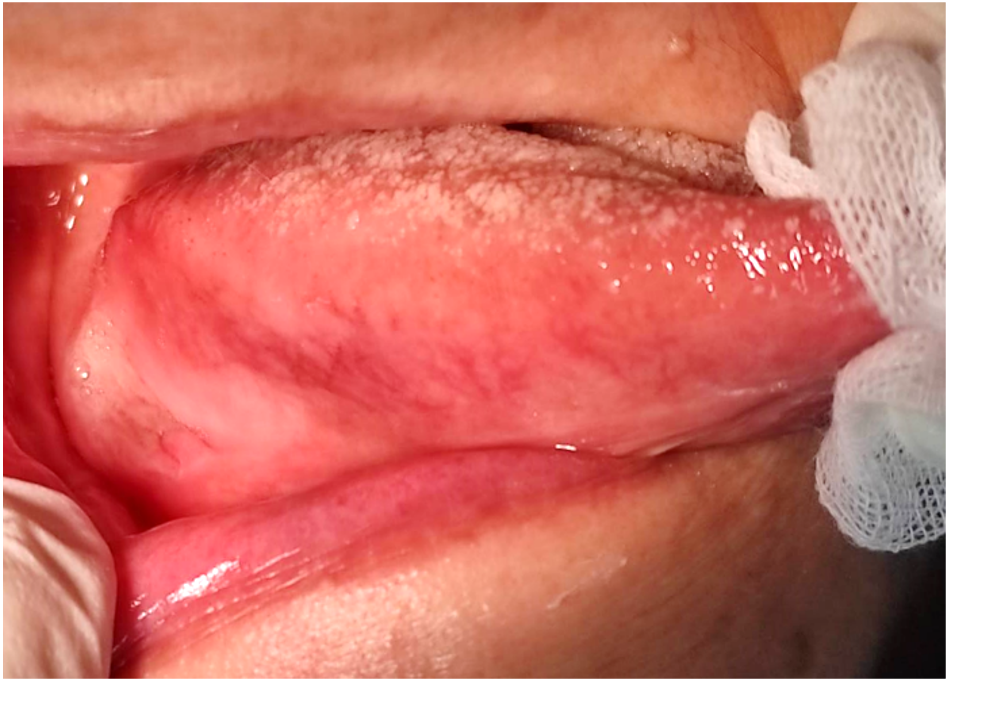

Supplement: Supplementary file 1 [file reports-09-00036-s001.zip › Supplementary Material_/Figure 7-1.png]

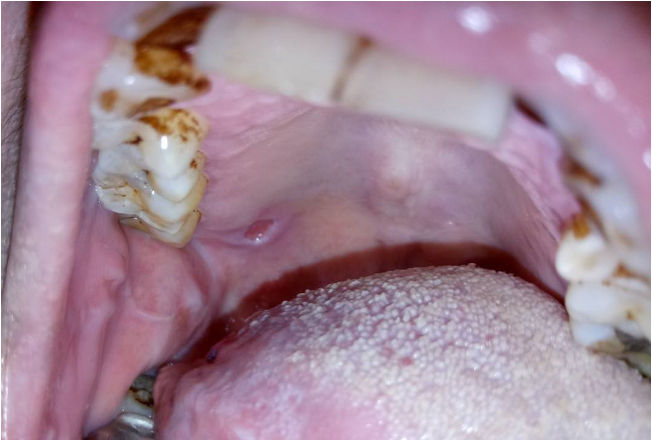

Supplement: Supplementary file 1 [file reports-09-00036-s001.zip › Supplementary Material_/Figure 7-2.png]

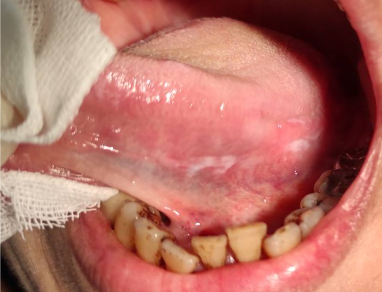

Supplement: Supplementary file 1 [file reports-09-00036-s001.zip › Supplementary Material_/Figure 7-3.png]

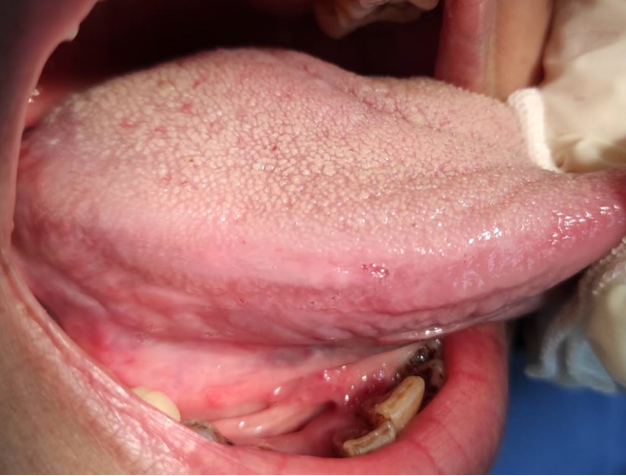

Supplement: Supplementary file 1 [file reports-09-00036-s001.zip › Supplementary Material_/Figure 8-1.png]

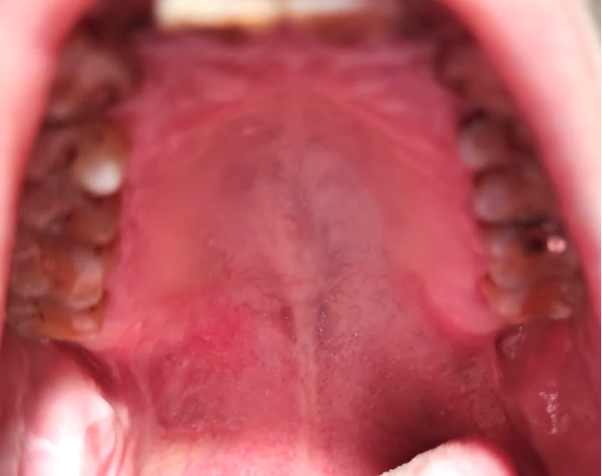

Supplement: Supplementary file 1 [file reports-09-00036-s001.zip › Supplementary Material_/Figure 8-2.png]

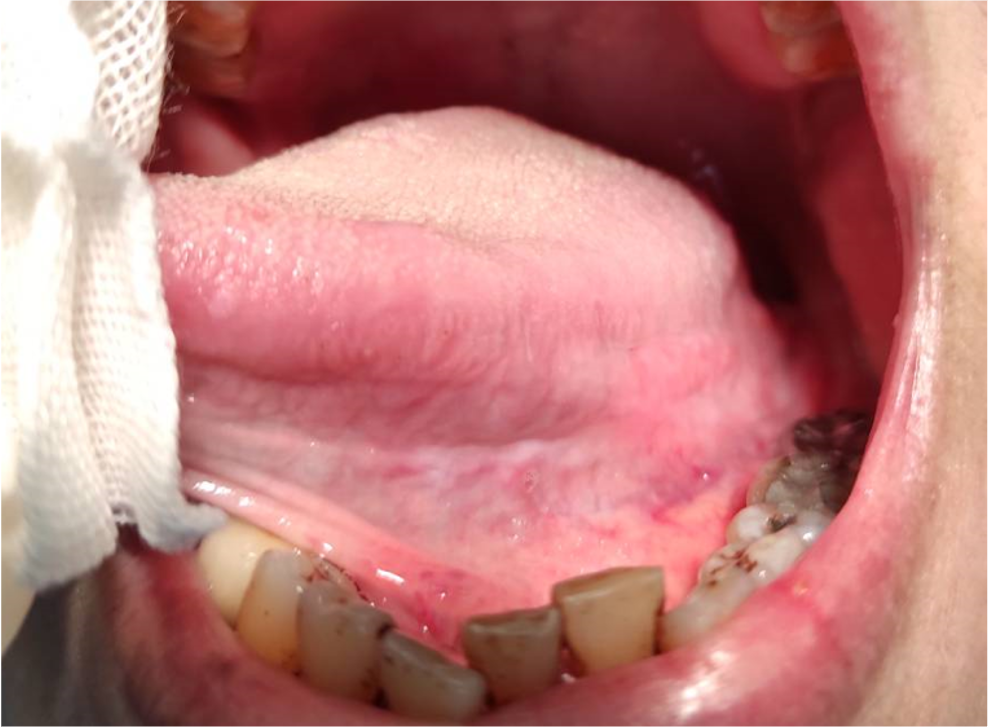

Supplement: Supplementary file 1 [file reports-09-00036-s001.zip › Supplementary Material_/Figure 8-3.png]

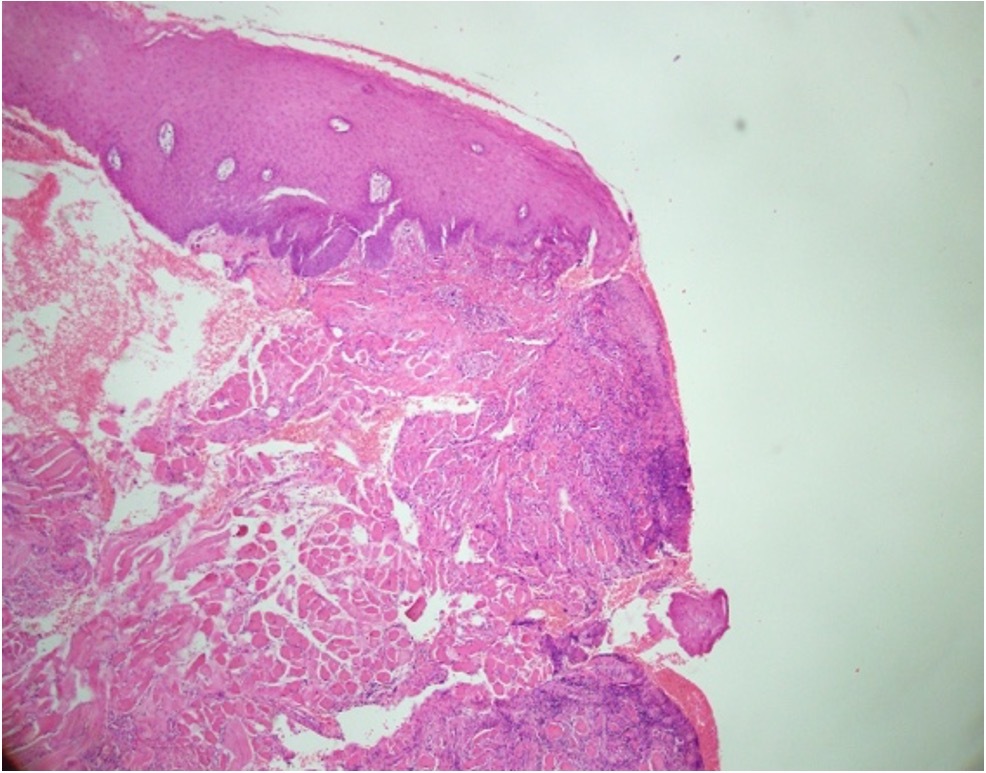

Supplement: Supplementary file 1 [file reports-09-00036-s001.zip › Supplementary Material_/Figure 9-1.jpg]

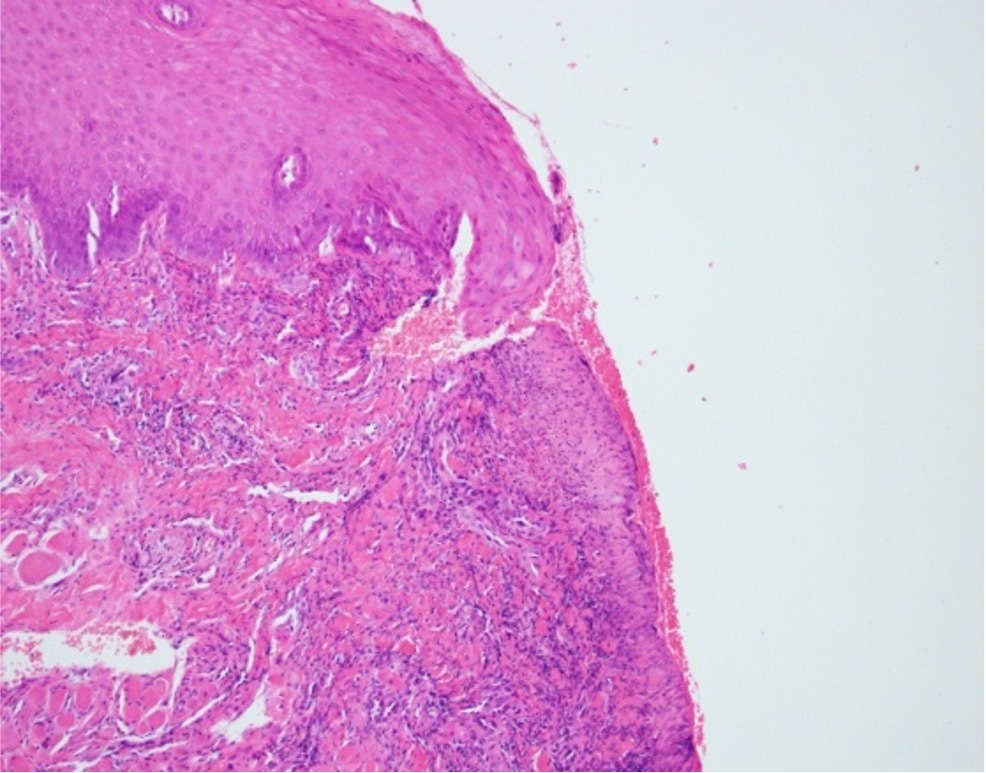

Supplement: Supplementary file 1 [file reports-09-00036-s001.zip › Supplementary Material_/Figure 9-2.jpg]

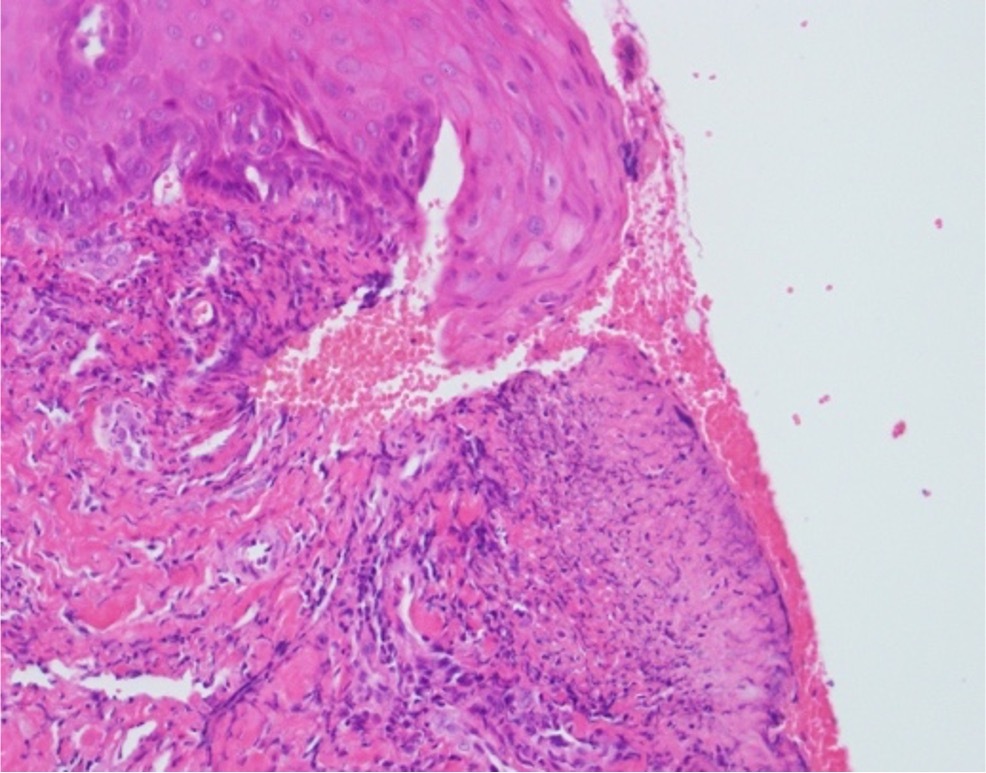

Supplement: Supplementary file 1 [file reports-09-00036-s001.zip › Supplementary Material_/Figure 9-3.jpg]
